# Impact of Training and Education Programs for Health Care Professionals on Video and Text-Based Meetings in Ensuring Health Care Quality: Protocol for a Scoping Review

**DOI:** 10.2196/69963

**Published:** 2025-07-09

**Authors:** Md Shafiqur Rahman Jabin, Aneekah Ashfaq, Nussrat Bi, Evalill Nilsson

**Affiliations:** 1 Department of Medicine and Optometry Linnaeus University Kalmar Sweden; 2 Faculty of Health Studies University of Bradford Bradford United Kingdom; 3 Faculty of Management, Law and Social Sciences University of Bradford Bradford United Kingdom

**Keywords:** chats, emails, health care professionals, patient encounters, text-based meetings, training and education, video conferences, video meetings, web portals

## Abstract

**Background:**

The use of video meetings and text-based meetings has surged and emerged as a critical tool in health care. These tools offer many benefits, such as patient prescreening, counseling services, remote patient tracking, and monitoring. With the increasing demand for technologies, health care professionals require training and educational competency development to sustain in the modern digital age. This necessitates synthesizing evidence about the existing training programs in arranging and regulating such meetings, the implementation, and reassurance about the effectiveness of these digital health meetings.

**Objective:**

The synthesis will also uncover what training programs for health care professionals to conduct video and text-based meetings are available, and if so, how they were implemented and their impacts from the perspectives of the organization, the staff, and the patients.

**Methods:**

The review will follow the Joanna Briggs Institute (JBI) methodology. The published studies will be searched in APA PsycInfo, PubMed, and CINAHL, and the unpublished studies through Mednar, Trove, OCLC WorldCat, Dissertations, and Theses. Studies published in English from 2003 will be considered. This review will include studies of health care professionals trained to communicate online with patients or service users, health care professionals, and health care organizations. The concept will involve online communication, such as conducting video and text-based meetings (emails, chats, and web portals), and the context will consider studies based on health care, hospitals or clinics, and primary care. A broad scope of evidence, including quantitative, qualitative, text, and opinion studies, will be considered. A total of 2 independent reviewers will screen the titles and abstracts and review the full text. Data will be extracted from the included studies using a data extraction tool developed for this study.

**Results:**

The results will be presented in a PRISMA-ScR (Preferred Reporting Items for Systematic Reviews and Meta-Analyses extension for Scoping Reviews) flow diagram. A draft charting table will be developed as a data extraction tool. The results will be presented as a “map” of the data in a logical, diagrammatic, or tabular form and a descriptive format. This protocol was first developed by the principal author at Linnaeus University in April 2022; however, a full search was undertaken in August 2024 as part of research development at the University of Bradford.

**Conclusions:**

The review will identify the knowledge gaps, clarify the concepts, examine emerging evidence, and thus make recommendations for future research on video consultation and text-based meetings.

**International Registered Report Identifier (IRRID):**

DERR1-10.2196/69963

## Introduction

### Overview

“Telehealth is broadly defined as the use of communications technologies to provide health care at a distance” [1]. The year 2004 witnessed a revolution in telehealth with the emergence of internet-based mobile technology. This development, which included mobile phones and personal digital assistants, made the Internet more accessible than ever before [2]. It provided cost-effective ways to access internet-based technology and facilitated 2-way communications from a broader range of locations, thereby democratizing telehealth practice [3].

In the wake of the COVID-19 pandemic and the evolution of telehealth with time, the role of digital technologies in health care has been amplified. The use of computers, the internet, and mobile phones has surged, enabling remote access through video meetings and other digital tools. This surge is a direct response to the pandemic’s social distancing and travel restrictions, underscoring the vital role of digital technology in maintaining health care services [4]. Along with the use of telehealth, additional forms of communication, including text-based meetings in the form of emails, chats, and web portals, have become essential to ensure that patient-related and other care-related activities can continue uninterruptedly in an unfamiliar environment [5,6]. After the COVID-19 pandemic, videoconferencing and text-based meetings have become a cornerstone of modern health care. Their easy accessibility, economic efficiency, comfort for patients, health care professionals, and the organization make them indispensable tools in the new health care landscape.

Video meetings have emerged as a critical tool in health care, facilitating family members’ virtual visits to the intensive care unit. This reduces the risk of infection and ensures social distancing, thereby enhancing patient safety [7]. These meetings can also help prescreen patients, for example, those who have flu symptoms and are required to be referred to a hospital, providing counseling services virtually, such as mental health [8] and behavioral health counseling [9,10], nutrition counseling, and weight management [11]. The patients can also be followed up after a hospital stay or surgery, requiring regular checks, patient monitoring, offering advance care directives, planning, and further counseling to patients and their families. This not only ensures continuous care but also reduces the need for frequent hospital visits, thereby minimizing the risk of infection [12,13]. This service application can even benefit social care, for example, by providing a bedside doctor’s visit to a nursing home, care home, or any assisted living residence without risking the patient or doctor spreading any infection [14].

The positive impact of digital technology on patient care is a reason for hope and optimism in the health care field. The improved patient outcomes may include behavioral telehealth competency improvement [15,16], behavior technician skills, patient engagement, skill acquisition [17,18], patient activation, and satisfaction [19]. Improved communication may involve connecting with patients virtually, breaking geographical barriers, and providing better accessibility for patients living in remote or underserved areas [19]. Such accessibility can minimize the need for extensive travel and enhance patient satisfaction with timely care and preventive care [16]. This powerful video consultation tool can also provide a platform for interdisciplinary collaboration among health care professionals, thus ensuring a streamlined health care process. This process may include a multidisciplinary approach, discussing complex cases, sharing insights, and making decisions for more comprehensive and patient-centric treatment plans [20,21].

With the increasing demand for technologies, tools, and applications, health care professionals are at the forefront of the digital transformation of health care. Their ability to effectively and efficiently practice medicine in the digital age is crucial [22-24]. Health care professionals, including nurses, physicians, specialists, clinicians, and practitioners, have a moral obligation to consider the consequences holistically, both intended and unintended, of digital health applications in routine clinical practice to uphold the oaths of providing patient care [25,26]. This demand further necessitates training and educational competency development for health care professionals to sustain in the modern digital age [10,27]. Developing digital health–related skills and competencies at any stage, particularly for conducting video meetings and conferences for health care professionals, is essential. The impacts of such training and education programs from the perspectives of patients, health care professionals, and the organization are of the utmost importance.

Such skills and competencies for health care professionals could include educational approaches, frameworks, or pedagogy related to understanding. This could also include applying, implementing, and evaluating the use and applications of video and text-based meetings at an individual, community, population, or society level to benefit human health and well-being. This would also answer the questions related to the ethical, legal, or social implications of health care personnel’s digital skills and competencies development. Emphasis will be placed on assessing and evaluating the effectiveness and cost efficiencies of those tools and applications associated with video conferencing and how they improve health care quality.

To shape this review, we will focus on health care professionals undergoing training and education programs to conduct video and text-based meetings with patients, such as emails, chats, and web portals. This evidence synthesis will inform us about the existing types of training programs in arranging and regulating such meetings, how they were implemented, and the results of the training from the perspectives of the organization, the staff, and the patients. This proposed scoping review aligns with the strategic direction of the NHS to understand the current trends and needs of health care professionals, patients, and service users. It can significantly improve the quality of health care services, potentially transforming the way health care is delivered [28]. Therefore, with their potential for widespread acceptance and adoption, the review results promise to meet today’s societal need, developing optimized guidance for providing information about personal and confidential patient information that can be used during a video consultation. Moreover, scoping reviews are designed to map key concepts and examine studies in a research area to provide an overview of the extent and nature of the current literature. This review aims to systematically identify and describe training programs for health care professionals to conduct online sessions with patients, making the scoping review methodology well suited to explore the available evidence without imposing strict inclusion criteria [29-31].

Despite a thorough search of reputable databases such as Campbell Systematic Reviews, the Cochrane Database of Systematic Reviews, PROSPERO, and Joanna Briggs Institute (JBI) Evidence Synthesis, no existing systematic reviews or scoping reviews on this specific topic were found. This underscores the unique and pioneering nature of our proposed scoping review, which promises to break new ground in digital health education.

### Aim and Review Questions

This scoping review aims to compile and synthesize the best available evidence regarding training health care professionals to conduct video and text-based meetings (emails, chats, and web portals) with patients, service users, health care professionals, and health care organizations. We would like to know whether there are any training programs out there and, if so, how they were implemented and what their impacts are from the perspectives of the organization, the staff, and the patients.

Specifically, the review questions are as follows:

1. What training and education programs are used to train health care professionals in conducting video and text-based meetings (emails, chats, and web portals)?
2. How are those training and education programs implemented to improve the quality of care and ensure health care quality?
3. What are the impacts of such training and education programs from the perspectives of patients, health care professionals, and the health care organization?

## Methods

### Overview

The proposed scoping review will be conducted following the JBI methodology for scoping reviews [32].

### Search Strategy

Databases will be searched for both published and unpublished studies. The approach to searching for studies for a scoping review will follow the standard 3-step method (Table 1).

**Table 1:** Inclusion and exclusion criteria.

Feature	Inclusion criteria	Exclusion criteria
Article type	Conference paper Gray literature Early access	—^a^
Language	English	—
Participants	Studies involving health care staff being trained to communicate online with service users Studies that include the implementation of training programs to train health care professionals to use online methods of communicating with service users	Studies that do not involve health care professionals (such as university students), and studies that pertain to training health care staff using video
Concept	Studies evaluate the impact of being trained to use online methods of communicating with patients on service users, health care professionals, and health care organizations	Studies that do not focus on the evaluation of the training impact
Year	Studies from 2003 through 2024	—
Context	Studies pertaining to health care, hospitals, or clinics, primary care	—

^a^Not applicable.

The first step will be an initial limited search of relevant databases, followed by an analysis of the title and abstract text words and the index terms used to describe the article. The search for published studies will include a 2-way search strategy. One is to search the journal and reference databases, such as PsycInfo, PubMed (this database interrogates by formatting it as “MEDLINE” via PubMed), CINAHL, and Web of Science. Another is to search article-based (journal) databases, such as ACM digital library, IEEE Xplore, and BMJ Journals. The search for unpublished studies will include Mednar, Trove, OCLC WorldCat, Dissertations, and Theses. A second search will be undertaken using all identified keywords (Textbox 1) and index terms across all included databases.

Additional search strategies, that is, citation search, specific researcher or article (eg, gold-standard article), and chain search, review reference list of the systematically selected articles, will be included to complement the search for published and unpublished papers. Studies, such as reviews (systematic, scoping, and umbrella) and letters to the editors, will be excluded. Any studies that lack ethical concerns will also be excluded. Studies published in English will be considered. Studies published from 2003 onward will be considered as the initial start for all searches to keep the dates consistent. Wireless internet-based technologies are of significant importance in the establishment of telehealth practices. To encompass more modern-day use of telehealth, this scoping review will consider research papers written in the early 2000s until the present. The collection of databases each had different publishing dates for their earliest research related to the keywords. CINAHL’s earliest relevant publication was published in 1990, whereas the earliest publication on APAPsycInfo articles was published in 2003, the latest publication out of the 4 databases used. To keep the dates consistent, 2003 was selected as the initial start for all searches.

**A list of keywords for the search strategy.**

**Participants**

- healthcare professional
- health worker
- healthcare staff
- healthcare provider
- clinician
- resident doctors

**Concept**

- video consultation
- video call
- telemedicine
- telehealth
- e-consultation
- text-based meeting
- email
- chat
- web portal
- online communication
- digital communication
- digital encounter
- training
- competenc*
- knowledge
- skill
- educational program*

**Context**

- healthcare
- hospitals
- clinics
- primary care

### Eligibility Criteria

This scoping review will include the following PCC (Population, Concept, and Context) framework. These mnemonics will be used as a guide (not policy); therefore, the inclusion criteria of this review will include a detailed description of types of participants, concepts, and context, as well as search strategies, data extraction, charting, analysis, and presenting the results. The eligibility criteria are listed and described in detail in Table 1.

### Participants

This review will include studies of health care professionals, workers, or providers trained to communicate online with service users, other health care professionals, and health care organizations.

### Concept

In this scoping review, the key concept is to evaluate the impact of online training methods of communicating, such as conducting video and text-based meetings (emails, chats, and web portals) with patients or service users, health care professionals, and health care organizations. The different formats of training will be considered (such as virtual vs in-person training and asynchronous modules vs workshops).

### Context

The scoping review will consider studies based on health care, hospitals or clinics, and primary care.

### Types of Sources

This scoping review will consider experimental and quasi-experimental study designs, including randomized controlled trials, nonrandomized controlled trials, before-and-after studies, and interrupted time-series studies. In addition, analytical observational studies, including prospective and retrospective cohort studies, case-control studies, and analytical cross-sectional studies, will be considered for inclusion. This review will also consider descriptive observational study designs, including case series, individual case reports, and descriptive cross-sectional studies for inclusion. Qualitative studies focusing on qualitative data will also be considered, including, but not limited to, designs such as phenomenology, grounded theory, ethnography, qualitative description, and action research. Text and opinion papers will also be considered since scoping reviews include a broad scope of evidence.

### Source of Evidence Selection

Following the search, all identified citations will be collated and uploaded into EndNote 20 (Clarivate Analytics), and duplicates will be removed. A pilot test, which is conducted to ensure the effectiveness and consistency of the screening process, will be carried out. Titles and abstracts will then be screened by 2 or more independent reviewers for assessment against the inclusion criteria for the review. Potentially relevant sources will be retrieved in full and their citation details will be imported into the JBI System for the Unified Management, Assessment and Review of Information (JBI SUMARI) [33]. A total of 2 or more independent reviewers will thoroughly assess the full text of selected citations against the inclusion criteria. Reasons for excluding sources of evidence in full text that do not meet the inclusion criteria will be recorded and reported in the scoping review. Any disagreements between the reviewers at each stage of the selection process will be resolved through open and inclusive discussion or with additional reviewers. The results of the search and the study inclusion process will be reported in full in the final scoping review and presented in the PRISMA-ScR (Preferred Reporting Items for Systematic Reviews and Meta-analyses extension for scoping Reviews) flow diagram [34,35].

### Data Extraction

A total of 2 or more independent reviewers will meticulously extract data from papers included in the scoping review using a data extraction tool they developed. The data extracted will include specific details about the participants, concept, context, study methods, and key findings relevant to the review questions, ensuring a comprehensive review. This rigorous process instills confidence in the reliability of the results.

A draft charting table, designed to be adaptable, will be developed as a data extraction tool. The charting table will be modified and revised as necessary while extracting data from each included evidence source, ensuring the review remains flexible and responsive to the data. This adaptability ensures that the review process is responsive to the nuances of the data, providing a comprehensive and accurate review.

We will extract variables to ensure alignment with our research questions, while some variables may emerge during full-text screening; therefore, it is essential to predefine the key data extraction elements (as the protocol is structured). Clearly outlining these variables will ensure consistency in data collection, enhance transparency in the scoping review methodology, and strengthen the study’s replicability. We will also follow the JBI Manual for Evidence Synthesis, which provides a basic data extraction table template to ensure completeness, methodological consistency, and adherence to best practices in data extraction [36].

## Results

The results will be presented as a “map” of the data extracted from the included papers in a logical, diagrammatic, or tabular (as necessary) form and in a descriptive format that aligns with the objective and scope of the review. Some critical information that the charting table will include are (but are not limited to): year of publication, country of origin for the study, aims, study population and sample size, methodology/methods, type of intervention/comparator, duration of the intervention, and type of outcomes and how they were measured (if applicable). A clear explanation for each category will be provided, accompanied by a narrative summary describing how the results relate to the review objective and questions.

The principal author first developed this scoping review protocol as part of research development at the University of Bradford, which has been undertaking a full search since July 2024. We expect the analysis to be completed by March 2025 and the final scoping review manuscript to be submitted by August 2025, providing a clear timeline for the review’s completion. This timeline ensures the audience is informed and prepared for the review process.

## Discussion

### Expected Findings

With the continuous rise of the application of digital technology, health care professionals have transformed their way of delivering patient care, collaborating, and following up on staying connected. Among all digital health care services, the most prominent advancement in recent years is the widespread usage of videoconferencing. This initiative (a scoping review) is a significant part of an international collaboration with the Region Kalmar Län, Sweden. Together, we aim to create a digital learning environment for training health care professionals to communicate with patients using only short text messages. Face-to-face workshops are crucial in this initiative, as they will enhance the digital learning environment, foster a sense of community, and promote the exchange of experiences among health care professionals. This collaboration will include patient and public involvement, arranged by the Faculty of Health Studies, University of Bradford, and already existing stakeholder involvement by the eHealth Institutes, Linnaeus University.

The improved patient outcomes may include behavioral telehealth competency improvement [15], behavior technician skills, patient engagement, skill acquisition [17], and patient activation and satisfaction [19]. Improved communication may involve connecting with patients virtually, breaking geographical barriers, and providing better accessibility for patients living in remote or underserved areas [19]. Such accessibility can minimize the need for extensive travel and enhance patient satisfaction with timely care and preventive care [16]. This powerful video consultation tool can also provide a platform for interdisciplinary collaboration among health care professionals, thus ensuring a streamlined health care process. This process may include a multidisciplinary approach—discussing complex cases, sharing insights, and making decisions for more comprehensive and patient-centric treatment plans [20].

Health care professionals need to be equipped with the skills to conduct virtual patient encounters. This involves understanding the ethical, legal, and social implications of digital health applications, particularly video and text-based meetings [22]. It also includes considerations of patient privacy and data security, the potential for increased access to care, and the impact on the doctor-patient relationship. It also involves understanding the potential for digital health to exacerbate existing health disparities or create new ones, as well as the ethical considerations of using digital tools to monitor and manage patient health. However, with time and resources constrained on the operative level in the busy sociotechnical health care environment, health care professionals cannot dedicate themselves outside their regular working hours. Their heavy workload also adds complexity to engaging and retaining skilled staff and experts within the health care organization [37]. Therefore, it is essential to set up an initial and ongoing training process on how to skillfully conduct virtual patient encounters skillfully, ideally in association with the vendors, and set aside sufficient paid time for health care professionals to be appropriately trained [28,38].

The internet, which was first used in 1983, gained popularity and usage in 1999 with the introduction of Wi-Fi on laptops and further in 2004 with the emergence of internet-based mobile technology. These milestones significantly increased the accessibility and reach of telehealth. The role of wireless internet-based technologies, such as mobile phones and personal digital assistants, in establishing telehealth practices is paramount, marking a significant shift in health care delivery [39,40]. This scoping review, aiming to capture the modern-day use of telehealth, will consider studies published from the early 2000s to the present. The significance of this review lies in its comprehensive coverage of the evolution of telehealth, providing valuable insights for health care professionals, researchers, and academics.

### Strengths and Limitations

The use of the PRISMA-ScR framework is specifically designed for scoping reviews as a reporting checklist to serve as a guideline to enhance the quality, transparency, and consistency of the entire scoping review process [41] (Multimedia Appendix 1). The findings of our review, while requiring caution due to our search limitations in language and publication period, are of significant value. These findings are the result of a transparent and comprehensive strategy, which includes the standard 3-step method and gray literature for additional insights [42]. The review is a crucial step in understanding the evolution of telehealth. Each database had different publishing dates for its earliest research related to the keywords. CINAHL’s earliest relevant publication was in 1990, while the earliest publication on APAPsych articles was in 2003, the latest among the 4 databases used. To maintain consistency, 2003 was chosen as the initial start for all searches, ensuring the relevance and timeliness of the research.

We acknowledge the possibility of a limited number of included studies, which may introduce bias, and we are committed to following the Evidence-based Practice Center (EPC) Methods Guide proposed by the Agency for Healthcare Research and Quality (AHRQ) to minimize this risk [43]. The scope of the review is of utmost importance, as it guides our search and interpretation of results. We must exercise caution, as most of the hits in the search may focus on training health care staff using video rather than training them on conducting video meetings with patients. It is also worth noting that articles on training health care staff in patient encounters may have been more prevalent during the pandemic (2020-2022) when digital encounters peaked.

We are committed to inclusivity to ensure the scoping review generates generalizable findings. To support the interpretation of findings and the dissemination of the review, we will hold discussions with health care professionals, patients, and the relevant public. Their perspectives are invaluable and will be integral to the review process.

### Conclusion

No current or underway scoping reviews on the topic were identified. This evidence synthesis, a collaborative effort involving researchers and practitioners, will inform us about the existing types of training programs for arranging and regulating online meetings, how they were implemented, and the training’s results from the perspectives of the organization, the staff, and the patients. This review will uncover the shreds of evidence requiring diligent attention to identify the knowledge gaps, clarify the concepts, examine emerging evidence, and thus make recommendations for future research on video consultation and text-based meetings.

## Appendix

Multimedia Appendix 1 PRISMA-ScR checklist.

## Abbreviations

AHRQ: Agency for Healthcare Research and Quality
EPC: Evidence-based Practice Center
JBI: Joanna Briggs Institute
JBI SUMARI: JBI System for the Unified Management, Assessment and Review of Information
PCC: Population, Concept, and Context
PRISMA-ScR: Preferred Reporting Items for Systematic Reviews and Meta-Analyses extension for Scoping Reviews

## Data Availability

All supporting data will be reported in the manuscript. The authors will make any raw data sets that support the results available as supplementary files.
